# Management of Oropharyngeal Dysphagia in Laryngeal and Hypopharyngeal Cancer

**DOI:** 10.1155/2012/157630

**Published:** 2012-12-31

**Authors:** Jose Granell, Laura Garrido, Teresa Millas, Raimundo Gutierrez-Fonseca

**Affiliations:** ENT Department, Rey Juan Carlos Hospital, Gladiolo, 28933 Mostoles, Spain

## Abstract

On considering a function-preserving treatment for laryngeal and hypopharyngeal cancer, swallowing is a capital issue. For most of the patients, achieving an effective and safe deglutition will mark the difference between a functional and a dysfunctional outcome. We present an overview of the management of dysphagia in head and neck cancer patients. A brief review on the normal physiology of swallowing is mandatory to analyze next the impact of head and neck cancer and its treatment on the anatomic and functional foundations of deglutition. The approach proposed underlines two leading principles: a transversal one, that is, the multidisciplinary approach, as clinical aspects to be managed in the oncologic patient with oropharyngeal dysphagia are diverse, and a longitudinal one; that is, the concern for preserving a functional swallow permeates the whole process of the diagnosis and treatment, with interventions required at multiple levels. We further discuss the clinical reports of two patients who underwent a supracricoid laryngectomy, a function-preserving surgical technique that particularly disturbs the laryngeal mechanics, and in which swallowing rehabilitation dramatically conditions the functional results.

## 1. Introduction

Dysphagia is defined as difficulty in swallowing. It is a symptom that expresses a disorder in the transport of food and endogenous secretions (saliva) through the upper digestive tract. Oropharyngeal dysphagia (OD) is a more anatomically restricted term referred to alterations in the transfer of the bolus from the mouth to the esophagus (that means, in bolus propelling from the mouth to the pharynx, in the pharyngeal reconfiguration during the swallow, or in the opening of the upper esophageal sphincter) [1].

OD is an inescapable concern in the management of patients with laryngeal and hypopharyngeal cancer. Being as a symptom at presentation, as an adverse effect during whatever the treatment, or as sequelae compromising the quality of life of the patients, swallowing disorders have to be adequately anticipated and dealt with [2]. Swallowing is one of the vital functions that the larynx is involved in. For an outcome to be considered functional, the patient has to be able to swallow in an effective and safe manner. Actually, preserving a functional deglutition is usually the most important goal of the different function-preserving surgical techniques on the larynx and the hypopharynx, as a larynx that does not prevent aspiration cannot be preserved.

 Even though OD has been specifically classified in the latest versions of the International Classification of Diseases, it has not yet been given the attention it deserves. OD is clearly underdiagnosed, and consequently under-treated, in spite of the high rate of complications it entails [3]. Although the highest prevalence corresponds to the elderly and patients with neurological disorders, head and neck cancer patients are a population where the disease directly affects the anatomy and function of the structures involved in deglutition. Therefore a neglect or inadequate management is inexcusable.

 The functional outcome of the patients with laryngeal and hypopharyngeal cancer will ultimately depend upon an accurate diagnosis, treatment, and rehabilitation. To accomplish breathing without a tracheostomy, oral feeding and close to normal phonation may become a difficult challenge. In many cases a function-preserving surgery will not be such without adequate rehabilitation. Moreover, chances of function-preserving surgery might be underestimated if a comprehensive swallowing rehabilitation protocol is not available. 

## 2. Physiology of Deglutition

### 2.1. Normal Swallowing

Normal swallowing is a complex series of neuromuscular events which has both reflexive and voluntary controls (the later making rehabilitation possible). Although swallowing is a smooth and continuous process, it is conventionally divided into three phases in which timing is controlled by different central pattern generators of the brainstem [4]. The *oral phase* is voluntary. The food enters the oral cavity and is mixed with saliva and masticated to form a cohesive bolus. Lips, tongue, teeth, mandible, and palate are involved in the preparation and propulsion of the bolus. The *pharyngeal phase* is initiated when the tongue pushes the bolus towards the posterior pharyngeal wall, eliciting a series of programmed responses: the soft palate elevates to prevent nasal reflux, pharyngeal constrictor muscles contract to push bolus through the pharynx, laryngeal sphincter mechanisms close to prevent aspiration (epiglottis inverts and true and false vocal folds adduct), the larynx is pulled in an anterior and superior direction, and so cricopharyngeal muscle (upper esophageal sphincter) is opened. The recent research in the physiology of swallowing has shown that this response can be modulated [5]. The *esophageal phase* is completely involuntary: peristaltic waves propel the bolus to the stomach.

Anatomic structures involved in deglutition have a complex motor and sensory innervation by the cranial nerves (CN). The trigeminal nerve (CN V) is responsible for the general sensation of the face and for the motor supply to the main muscles involved in mastication. Facial nerve (CN VII) gives motor function to the lips and taste to the anterior two thirds of the tongue. The glossopharyngeal nerve (CN IX) gives general sensation to the posterior third of the tongue and motor supply to the pharyngeal constrictor muscles. The vagus nerve (CN X) gives motor function to the soft palate, pharynx, larynx and esophagus, and general sensation to the larynx. This includes the lingual side of the epiglottis, and important sensory site that always triggers deglutition (a basic mechanism to protect the airway). Finally, the hypoglossal nerve (CN XII) controls most of the muscles involved in tongue motility.

### 2.2. Swallowing in Head and Neck Cancer Patients

OD may be caused by anomalies involving the oral cavity, pharynx, and larynx that can be either structural or functional. Both head and neck cancer and its treatment have potentially devastating effects on swallowing. The site of the primary tumor will determinate symptoms due to alterations of different phases of deglutition, sometimes similar to those that will have to be managed after the treatment. Although we focus our interest on the larynx that has certainly a central critical role in deglutition, an isolated discussion on it would be absurd, as patient situation is usually more complex: there are different factors conditioning OD, locally advanced tumors will extent to neighboring sites, the neck will have to be treated in many instances, and treatment schemes are usually mixed. In general, lesions in the oral cavity will impair bolus preparation, containment, and posterior movement of the bolus to the pharynx. Pharyngeal and laryngeal lesions may show variably altered swallow responses that may condition laryngeal penetration or even tracheal aspiration.

The three modalities of treatment used for head and neck malignancies, radiation therapy, chemotherapy, and surgery will have different effects on swallowing that can be additive. *External-beam irradiation* has early effects due to mucositis that may cause superficial ulceration and pain. Although mucositis may condition reliance on nonoral nutrition, it will usually be temporary. Late effects are related to xerostomia and scars. Xerostomia is the most usual complain and will last for years [6]. Fibrosis due to radiation therapy may be expressed as strictures, sometimes requiring dilation or even surgery, muscle changes, and different mechanical changes that will affect deglutition: fixation of the hyolaryngeal complex, reduced tongue motion, and insufficient glottis closure…. There are some methods to prevent or minimize radiation sequelae, like shielding or modified radiation protocols (like intensity modulation). In general, irradiated patients will show reduced oral and pharyngeal functions, with longer transit times, more pharyngeal residue, and reduced cricopharyngeal opening time. Actually, postoperative radiation therapy seems to be the main factor influencing worse functional results after partial surgery of the larynx [7].

*Chemotherapy* will also have two types of adverse consequences on swallowing. The first one is also related to mucositis. Virtually all of the patients on chemotherapy schemes for head and neck cancer will show some degree of mucositis. It will be clinically significant in up to 40% of the patients and in 100% of those under chemoradiation [8]. On the other hand, patients may have nausea and vomiting along with extreme weakness that might impair swallowing.

Effects on swallowing caused by *surgical treatment* are of particular interest because it is precisely the severity of those that will allow the surgery to be considered a function-preserving one. The goal is preserving function with oncological warranties. Effective and safe swallowing is a requirement for oral alimentation, and so it is one of the main objectives pursued by the different surgical procedures. In general, surgical treatment will alter the structure and function of different anatomical sites in the oral cavity, pharynx, and larynx. In many cases structure can be restored in a close-to-normal anatomic way by different reconstructive procedures. Normal motion and sensation are far more difficult to achieve.

Treatment of tumors of the *oral cavity* will cause a range of predictable problems depending on its location, size, and type of reconstruction performed. The function of the oral (labial) sphincter may be affected by local resection or by lesions of the marginal branch of the facial nerve. If soft or hard palate are involved, nasopharyngeal reflux might occur. Resections of the floor of the mouth may lead to the loss of the glossoalveolar sulcus or fixation of the tongue. It is usual to close defects on the oral cavity with flaps. This may cause a variety of problems: they may obstruct bolus passage if they are too bulky, they have no motor function and so there might be a loss of propulsive force, and as they are usually nonsensate flaps, they will lack the normal sensation required to guide the bolus to the oropharynx. Tumors affecting the tongue base are probably the ones in this region most prone to cause dysphagia, as tongue base is critical in initiating swallow, propelling the bolus through the pharynx, and obtaining an efficient pharyngeal peristalsis. Therefore pharyngeal stasis might occur, posing the risk of postswallowing aspirations. Excision of the tongue is particularly problematic. Resections of up to one third of the tongue are well tolerated; also preserving some neural control (and thus some movement, at least in one side) is critical. If the tongue gets fixed to the floor of the mouth or the hypoglossal nerve is damaged, dysphagia will worsen, as it will be difficult to control and direct the bolus inside the mouth, to chew, and to propel the bolus posteriorly. Total glossectomy is a particularly difficult situation to achieve oral nutrition.

Resection of different segments of the *pharynx* might impair pharyngeal peristalsis. It might also cause laryngeal fixation with risk of laryngeal penetration or aspiration.

**Figure 1:**
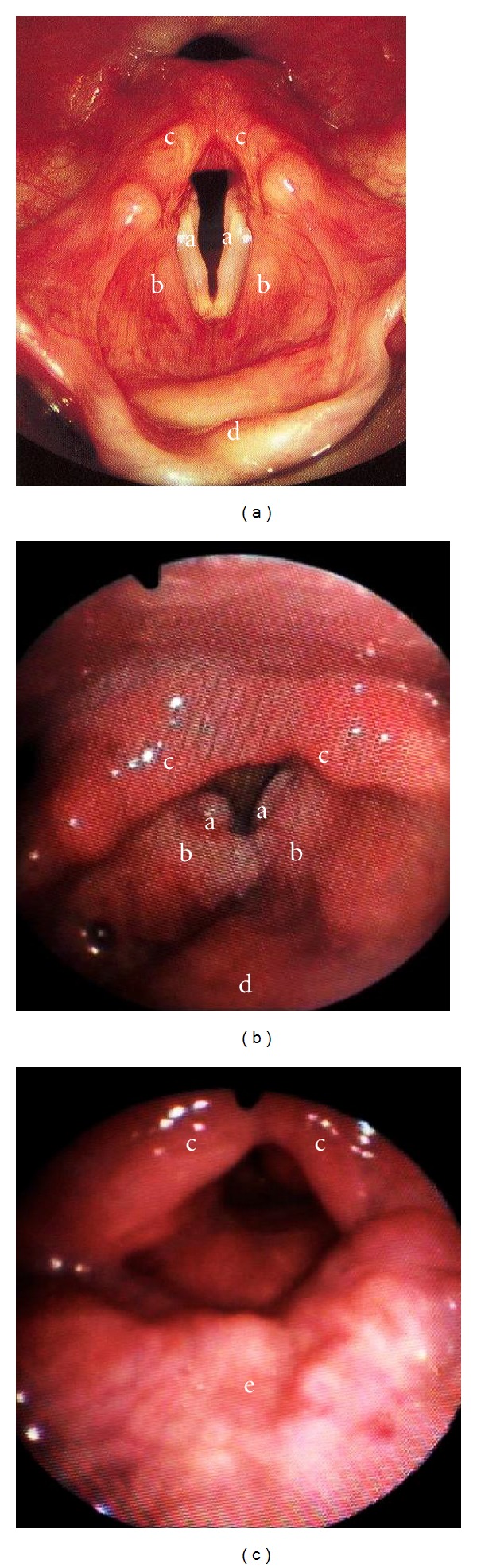
Anatomy of the laryngeal sphincter. (a) The normal larynx provides a number of intrinsic and extrinsic mechanisms to protect the airway during the swallow. (b) Glottic tumors affect the main sphincter of the larynx, the vocal folds. (c) After a supracricoid laryngectomy with cricohyoidopexy most of the sphincters are lost (including the vocal folds and the epiglottis), and the airway is closed by displacement of the arytenoids against the tongue base. a: true vocal folds. b: false vocal folds. c: arytenoids. d: epiglottis. e: tongue base.

The primary and evolutionarily original function of the *larynx* is related to the fact that there is an aerodigestive confluence and thus a need for a swallowing act that guides the food in the right direction. The larynx is elevated and moved anteriorly while its sphincters close, thus preventing food to enter the airway. Also this excursion will help open the cricopharyngeal sphincter. The main risk of partial laryngeal surgery is aspiration. The more altered the security mechanisms, the higher the risk for complications. A clear example is supracricoid laryngectomy where just a sketch of a sphincter remains [9]. Surgical techniques are designed to prevent this complication. Supraglottic laryngectomy will alter some of the sphincter mechanisms of the larynx, and possibly laryngeal excursion during swallowing. In the classic open surgery this late problem (and partially the former one) is minimized by laryngeal suspension [10]. Transoral laser surgery causes a much smaller damage to the extrinsic mechanism involved in laryngeal movement, and even though the technique does not in any way “suspend” the larynx, swallowing in the postoperative period tends to be much better [11]. In extended procedures involving portions of the tongue base, hyoid bone, and others, swallowing prognosis might be worse. Supracricoid laryngectomies, a more aggressive type of partial horizontal laryngectomy, pose problems on the airway (and thus on the possibility of decannulation), on phonation and on swallowing, that may vary depending on every particular case (Figure 1). Nevertheless, with a careful preoperatory patient selection functional results can be outstanding [12]. Vertical hemilaryngectomy requires and increased effort for laryngeal adduction and frequently facilitating maneuvers, as one side of the larynx in loss and reconstructed with more or less static structures. Patients undergoing total laryngectomy usually do not have significant swallowing problems after surgery (although they usually do have at diagnosis, so frequently from the patient's view in this regard function is improved). Occasionally they may have problems with bolus propelling or strictures in the pharyngeal suture, as well as alterations in the cricopharyngeal sphincter and esophageal motility [13].

## 3. Diagnosis of Dysphagia

### 3.1. General Approach to Patients with Swallowing Disorders

OD has a high prevalence in the general population [14]. It is associated with a rate of morbimortality and impairment in the quality of life. It has a maximum incidence in the elderly, patients with neurological disorders and patients with head and neck cancer. Patients in these and other clinical situations may be candidates to be evaluated for swallowing disorders. There is growing evidence that screening for dysphagia is advisable in different groups of patients [15]. A high percentage of patients with head and neck cancer, and virtually all of those with advanced stage tumors, will suffer dysphagia before, during, or after treatment.

The assessment of OD should include a detailed history focusing on the medical status of the patient, and a comprehensive clinical examination. From the clinical point of view, although the efficiency of oral nutrition might be impaired (which would lead to malnutrition and dehydration if no alternative route is used), it is the possibility of aspiration what focuses the most pressing interest. Usually patients with impaired safety of the swallow show cough (and ultimately aspiration and pneumonia), but silent aspiration is not unusual. Swallowing trials may be performed on a bedside fashion. The accuracy of the *volume-viscosity swallow test* for clinical screening of OD and aspiration has been demonstrated [16]. It assesses a series of items on the effectiveness and safety of the swallowing in a systematic fashion with different volumes (5, 10, and 20 mL) and viscosities (liquid, nectar, and pudding). Also silent aspiration might be suspected but needs to be confirmed with further research.

The main goals of the clinical assessment are screening for the presence of dysphagia, and if so, determining the risk of aspiration and the feasibility of oral nutrition, therefore avoiding the most usual complications, namely, malnutrition and aspiration pneumonia. Modifications of the diet, facilitating maneuvers, and other therapeutic measures might be recommended, but if efficiency and safety are not warranted, nonoral nutrition should be advised.

Finally, dysphagia-related quality of life of the patients can also be assessed by specific questionnaires [17]. This will give the patients' view on functional results regarding swallowing. Virtually every patient with head and neck cancer will show some degree of at least temporary dysphagia that will impact his quality of life. Also some conditions associated with the swallowing disorder, like requiring a nasogastric feeding tube, are felt as particularly troublesome for the patients [18].

### 3.2. Instrumental Diagnostic Tests

Instrumental assessment of swallowing provides useful information on both the structure and function of the swallowing mechanism, also when anatomy has been changed by the surgical procedure. There are two main diagnostic procedures to be used in the assessment of OD.

**Videofluoroscopic Assessment of Swallowing**

**Figure 2:**
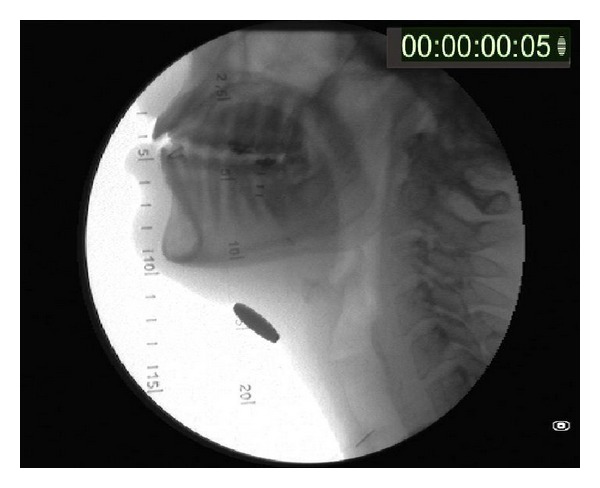
The anatomy of the upper aerodigestive tract is clearly identified in lateral cervical plain radiographs. Note the silhouette of the hard and soft palate, the tongue, including the tongue base, and the vallecula, and the epiglottis; the situation of the glottis can be easily estimated. The reference for the measurement of the distances is a 10 cents of eurocoin applied with adhesive tape to the midline of the neck (a constant size in any possible position: 20 mm), and there is a timer in the top right corner counting by the hundredth of a second.

**Figure 3:**
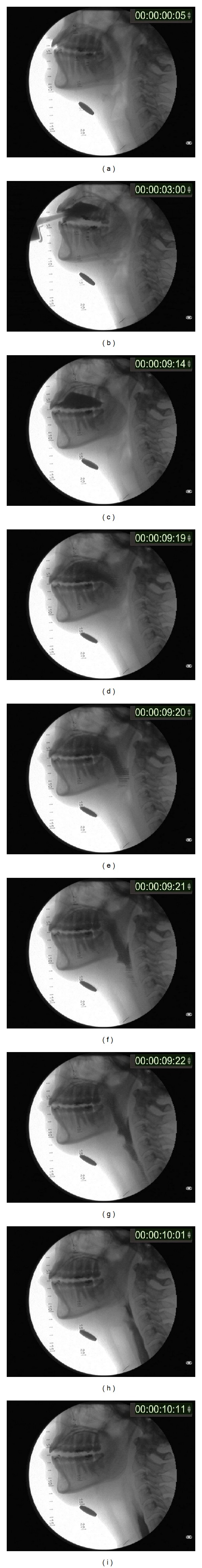
Normal swallowing. The sequence of the phases of deglutition is demonstrated in still pictures of a videofluoroscopic examination. (a) Normal anatomy at rest. (b) The contrast is introduced in the mouth with a syringe. (c) Oral phase, bolus preparation. (d) Bolus posterior propulsion. (e) The pharyngeal phase is triggered when the bolus enters the oropharynx. (f) Laryngeal closure. (g) Opening of the upper esophageal sphincter. (h) and (i) Esophageal phase. Note that it takes less than a second to complete the transit of the bolus through the pharynx.

It is the most widely used diagnostic test. A high-resolution video is used to record a movie that can be later measured and timed on slow motion or static pictures (Figure 2). The modified barium swallow is the technique of choice for the diagnosis of oropharyngeal dysphagia when there is an attributable cause (as it does happen in head and neck cancer patients) [19]. The test allows the examiner to observe the interactions between the different swallowing phases and assesses the whole dynamics of the process (Figure 3). The benefit of the different swallowing strategies might be also assessed. The main drawback is that the technique uses ionizing radiation.

**Fiberoptic Endoscopic Evaluation of Swallowing**

**Figure 4:**
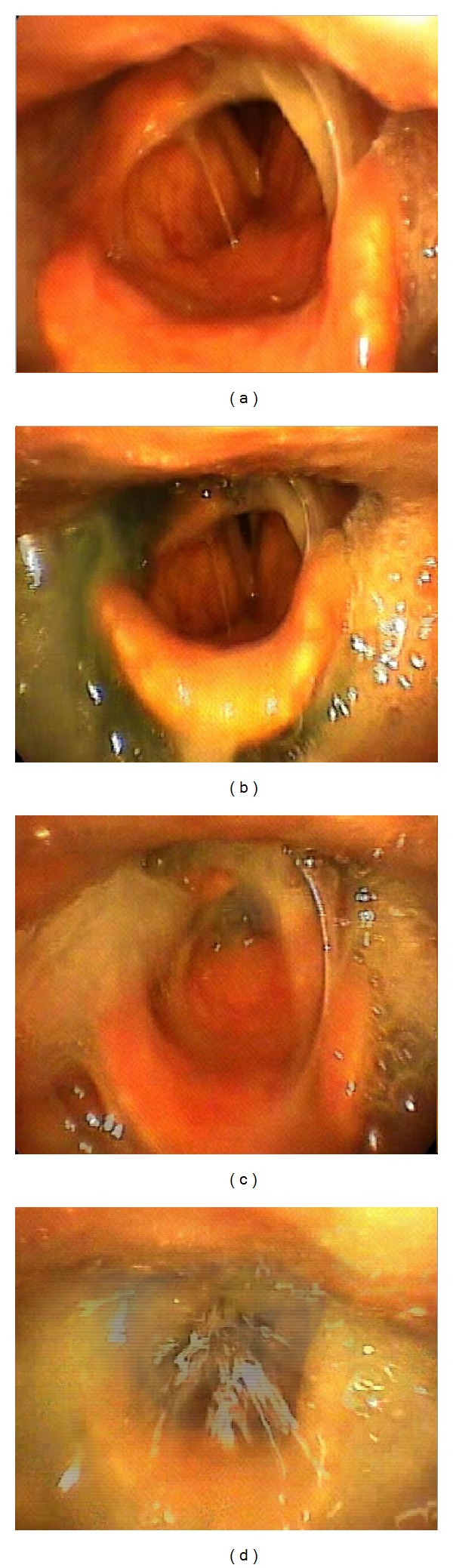
Fiberoptic endoscopic evaluation of swallowing. (a) Anatomy and function can be evaluated at the beginning of the examination. Endogenous secretions retention and even penetration or aspiration at rest suggest sensory impairment. (b) The swallow trial always starts with honey consistency, the easier to manage. Dysfunction is further corroborated as there is immediate penetration. (c) The patient shows evident aspiration. (d) The larynx is extraordinarily dysfunctional from the safety point of view. Obviously the patient has to refrain from oral alimentation.

It is a way of directly observing the act of swallowing by means of a flexible endoscope passed through the nose and situated at the nasopharynx facing down towards the hypopharynx. Different consistencies and volumes of a colored substance are used for the test. In the swallow test both oral and pharyngeal phases will be evaluated. The examination provides particularly complete information on the structure and function of the pharyngeal phase. It can also assess palatal function and the normal movement of the larynx in respiration and phonation. First the bolus should be kept in the mouth. Dribble will indicate lip incompetency, and dripping to the hypopharynx will show incompetency of the palatoglossus closure, posing a risk of predeglutition aspiration. When asking the patient to swallow, tongue base movement should be observed to assess propulsion. There might remain residues in the mouth or exist nasopharyngeal reflux if there is nasopharyngeal incompetency. The sequence and synchrony of the movements of the pharyngeal phase should be observed, as well as eventual penetration (food enters the laryngeal vestibule but remains over the glottic plane) or aspiration (there is food in the airway under the vocal folds) (Figure 4). A sensitivity test can also be performed either with the tip of the endoscope or, ideally, with air pulses [20]. Stimulating the aryepiglottic fold will provoke medialization of the ipsilateral vocal cord. It also gives valuable information on the management of secretions, and it can also be used as a feedback in retraining therapy.

A number of other procedures can provide additional information on selected cases: other endoscopic procedures like conventional upper digestive tract endoscopy or transnasal esophagoscopy, esophagic manometry, 24-hour pH-metry….

## 4. Treatment

Historically, the systematic therapeutic approach to patients with OD was first attempted by speech pathologist, as they realized that they could not treat their patients with cerebral palsy if patients were not able to adequately manage oral secretions [21]. The particular organization and composition of the therapeutic team may differ in every institution although a multidisciplinary team will be required, ideally in specific units devoted to the treatment of dysphagia [22]. Most of the swallowing disorders can be improved or solved with an adequate personalized training depending on the patient's condition.

The main goal of swallowing rehabilitation is to establish an effective and safe deglutition. This means that the patient can rely on oral diet and will not have aspiration. Patients should be informed before the oncologic treatment on the possibility of dysphagia and on the eventual need for rehabilitation. Although dysphagia is a usual symptom at diagnosis (and depending on the tumor it may improve with the treatment), pretreatment counseling on the expected swallowing difficulties will help prepare the patient for rehabilitation.

Some prophylactic measures may be undertaken. When radiation therapy is to be given, it is useful counseling on oral hygiene, avoidance of alcohol and tobacco, hydration, and artificial saliva when required. Chemotherapy-related mucositis would be given a symptomatic treatment, usually in the form or mixtures for mouthwashes. Severe mucositis can require hospitalization and intravenous treatment or even modifications of the scheme of treatment. When planning a function-preserving surgery, the surgeon has to make certain that, with the preserved or reconstructed structures, deglutition (without aspiration) will be possible with adequate rehabilitation. Otherwise, alternative options should be considered.

A detailed description of treatment modalities for swallowing impairment is beyond the scope of this paper. Nevertheless a brief description will be given. There is a wide array of therapeutic medical procedures that will fall in some of the following categories.

### 4.1. Adaptation Strategies

**Modifications of the Environment**

The patient will need a quiet environment and enough time to eat. Different specific instruments to introduce the food in the mouth may be preferable in different situations, including specifically designed ones like the “glossectomy spoon.” Although supervision is adequate, the patient should be encouraged to self-feeding (when possible).

**Diet Modifications**

Volume and consistency of the bolus should be modified according to the findings in the clinical tests. Food with a homogeneous consistency is preferable. Thickeners are a frequent and useful resource. Sour bolus has been found to significantly shorten pharyngeal transit time in patients with head and neck cancer [23].

**Orofacial Prosthetics**

They are in some cases an alternative to incompletely reconstructed or dysfunctional structures. The classical one is the obturator for palatal defects to prevent nasal reflux.

### 4.2. Swallowing Rehabilitation

#### 4.2.1. Indirect

**Muscular Rehabilitation**

Different physiologic exercises may be advisable, like motion exercises or resistance exercises for the jaw, lips, oral tongue, tongue base, laryngeal elevation, laryngeal closure….

**Sensory Procedures**

Sensory procedures enhance sensory feedback when it is impaired. A variety of choices are available: thermal stimulation by altering food temperature, tactile stimulation by applying pressure to the tongue, sensory stimuli (anterior facial arch) to elicit the oropharyngeal phase, introducing mastication (when possible)….

#### 4.2.2. Direct

**Postural Changes**

Postural strategies try to help the bolus flow in the desired direction. They also allow the patient to voluntarily modify the dimensions and relationships of the different anatomic structures. This may be used alone or in combination.

- *Chin-to-chest maneuver*: holding the chin down against the chest facilitates the contact of the tongue base with the posterior pharyngeal wall. It will also open the vallecula, and helps protecting the larynx from aspiration. It is advisable whenever there is a delay in the swallowing reflex.
- *Head extension*: helps nasopharyngeal closure and facilitates oral and pharyngeal transit when there is a deficit in the lip or nasopharyngeal closure, or impaired lingual propulsion. Adequately preserved laryngeal closure and elevation are imperative to prevent aspiration during the maneuver.
- *Head rotation*: to one side helps the bolus pass down through the opposite pyriform sinus and closes a damaged pharynx or a paralyzed larynx.
- *Head tilt*: makes gravity help the bolus down through the healthy side.
- *Lying supine or lateral*: minimizes the effect of gravity in the bolus when there is poor voluntary control of the mouth to pharynx passage.

**Specific Swallowing Maneuvers**

Swallowing maneuvers are designed to alter the physiology of the swallow.

- *Supraglottic swallow*: closes the vocal folds before and during the swallow to prevent aspiration. This is obtained by a voluntary apnea before the swallow. A voluntary after-swallowing cough is advised for any eventual silent aspiration. It is indicated when swallow reflex or glottic closure are delayed.
- *Effortful swallow*: augments voluntary contraction of the tongue and pharynx. It is useful when there is weakness in the tongue base or an altered pharyngeal peristalsis. It can be assisted by applying the hand on the patient's forehead and instructing him to press while swallowing.
- *Super-supraglottic swallow*: is an “effortful supraglottic swallow.” It is used when laryngeal closure is deficient.
- *Mendelsohn maneuver*: enhances anterior-superior displacement of the larynx to facilitate cricopharyngeal opening. It is performed by manual displacement and holding of the larynx, and it is indicated when the normal physiologic excursion is impaired or when deglutition is uncoordinated. It improves the transit of the bolus and reduces residues.
- *Masako maneuver*: (tongue holding maneuver by biting it) facilitates the movement of the tongue base and its contact with the posterior pharyngeal wall.
- *Repeated swallow*: “dry swallow” reduces residues.

**Figure 5:**
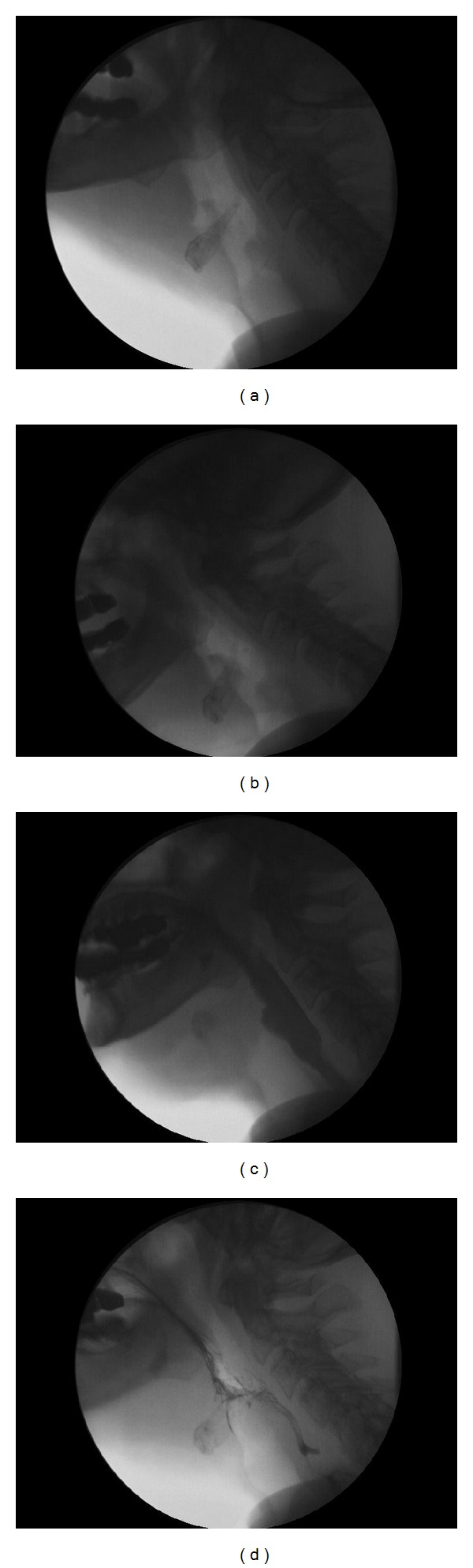
Case report 1. Male, 51. Right vocal fold epidermoid carcinoma with impaired mobility extending to the anterior commissure. The patient received a supracricoid laryngectomy with cricohyoidoepiglottopexy. A late 6-month videofluoroscopic control is shown. (a) Postsurgical anatomy at rest. The distance between the hyoid bone and the trachea is reduced while vocal folds are missed. The new laryngeal sphincter is sensibly shorter in the anterior-posterior direction. (b) Chin-to-chest maneuver to start the swallow (notice the contrast in the oral cavity). (c) The swallow is effective. (d) Although there is some residue, the patient shows no aspiration (the swallow is safe).

**Figure 6:**
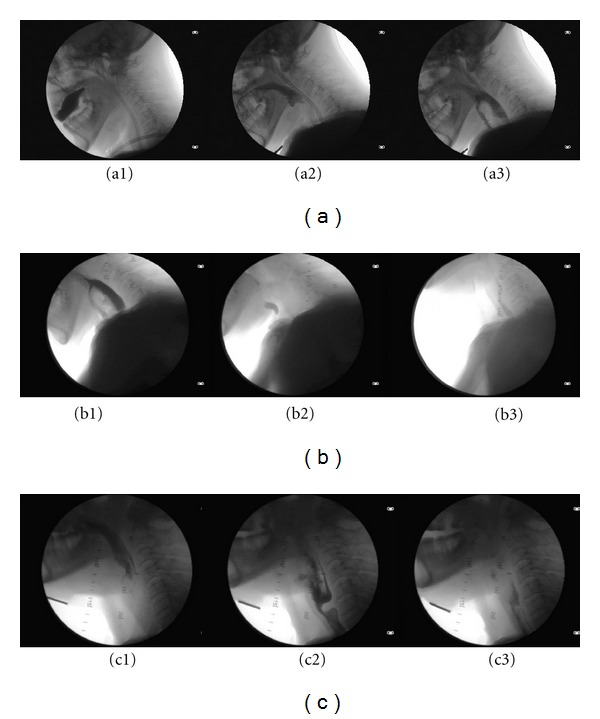
Case report 2. Male, 64. T2 bilateral glottic epidermoid carcinoma affecting the right laryngeal ventricle and with limited extension to the subglottis. He received a supracricoid laryngectomy with cricohyoidopexy and bilateral functional neck dissection. The sequence of postoperative videofluoroscopic examinations is presented. (a) Early postoperative (10 days) with noticeable aspiration (a3). The patient received a temporary gastrostomy and was instructed in swallowing maneuvers (chin-chest, supraglottic swallow, effortful swallow, and repeated swallow), and was advised to do exercises with honey-pudding consistency. (b) In a 3-month videofluoroscopic control there is penetration (b1) with residue in the laryngeal vestibule (b2) that is cleared by voluntary coughing and repeated swallow (b3). (c) Three months postop: the larynx and the trachea are free of alimentary contents. Note the typical cricopharyngeal bar (c2). Deglutition is effective and safe (c3).

Depending on the surgical procedure, and on the swallowing alterations observed in the clinical evaluation, patients will require a personalized therapeutic program that will include a number of the abovementioned resources (Figures 5 and 6).

There should be an additional topic for the surgical treatment of OD. We would just remark in this regard two different perspectives. The first one is the importance of a meticulous care in the technique of the function-preserving surgery, with particular attention in the surgical steps specifically directed to improve or preserve swallowing. This is of course critical in the most disturbing procedures, like supracricoid laryngectomies [24]. The other one is the surgical treatment of different clinical situations causing OD. There are a number of defined entities with specific surgical treatment like procedures for vocal cord medialization.

There are also nutritional concerns in the treatment of patients with head and neck cancer, not only before treatment, but also afterwards [25]. Swallowing alterations will put the patient on higher risk for malnutrition. Patients with mucositis, xerostomia, dysgeusia, odynophagia, or those on liquid diet might be unable (or unwilling) to meet their nutritional requirements. Nutritional support will improve functional outcomes and the patient's sense of wellbeing.

Finally, if oral nutrition is not possible, an alternative method of enteral nutrition should be offered. Nasogastric tube is a temporal measure (i.e., for the postoperative period). When a long-term need is expected, gastrostomy should be the option taken. Sometimes the patient keeps on suffering aspiration even after oral nutrition withdrawal. In this situation the airway needs to be protected; this may be achieved by a tracheostomy with a cuffed cannula (although deglutition will be further impaired with this measure) or by means of laryngeal exclusion (usually by laryngectomy), which would of course destroy the expectations of a functional treatment, but would perhaps save the life of the patient.

## 5. Conclusion

Oropharyngeal dysphagia is a critical concern in any function-preserving surgical procedure in patients with laryngeal and hypopharyngeal cancer. Effectiveness and safety of swallowing have to be proved before reintroducing oral nutrition. This may be done either by clinical or instrumental methods, depending on every particular situation. There is a wide array of resources for swallowing rehabilitation when it is required. Swallowing rehabilitation is imperative in most aggressive procedures, to the extent that the functional outcome may rely on it.
